# Relationship Between Body Image and Women’s Mental Health During Pregnancy

**DOI:** 10.7759/cureus.104889

**Published:** 2026-03-09

**Authors:** Hiromi Matsufuji, Asuka Kuratomi, Subaru Ikeda, Seiichi Morokuma

**Affiliations:** 1 Department of Health Sciences, Kyushu University, Fukuoka, JPN

**Keywords:** anxiety, body appreciation, body image, depression, mental health, perinatal

## Abstract

Introduction

Mental health problems during pregnancy negatively affect the health of both women and infants. Although body image is considered one of the mental health factors, studies investigating the association between positive body image and mental health during pregnancy are scarce. The aim of this study was to assess the association between positive body image and mental health in the second trimester of pregnancy among Japanese women.

Methods

A cross-sectional study using an internet-based questionnaire was conducted among women during the second trimester of pregnancy. Positive body image and mental health were evaluated using the Japanese version of the Body Appreciation Scale-2 (BAS-2) and the 6-item Kessler Psychological Distress Scale (K6).

Results

Of the 468 women included in the analysis, 277 (59.2%) and 67 (14.3%) women showed K6 cut-off values of ≥5 and ≥13, respectively. A higher score in BAS-2, indicating a more positive body image, was associated with a lower risk of psychological distress (adjusted odds ratio (OR), 0.65; 95% confidence interval (CI): 0.50-0.84) and serious mental illness (adjusted OR: 0.68, 95% CI: 0.47-0.97). The associations were highlighted among women who overestimated their body size (psychological distress, OR: 0.43, 95% CI: 0.25-0.77; serious mental illness, OR: 0.18, 95% CI: 0.07-0.45).

Conclusion

Overall, positive body image was associated with a low risk of psychological distress and serious mental illness during the second trimester of pregnancy. These findings suggest that positive body image may influence improvements in mental health during pregnancy.

## Introduction

Mental health during pregnancy is an important public health concern. The pooled prevalence of any depression and major depression during pregnancy was 20.7% and 15.0%, respectively [1]. Antenatal depression and anxiety were associated with increases in low birth weight and preterm birth [2,3]. Additionally, antenatal mental illness predicts negative outcomes in postpartum psychological health and breastfeeding practice [4]. Working status, social support, smoking status, experience of violence, and marital status have been identified as influencers on mental health [1]. In addition, body image has been indicated as one of the important predictors of prenatal mental health problems [5].

Negative body image is considered similar to body dissatisfaction, a state in which individuals are dissatisfied with some parts of their body or overall appearance [6]. On the other hand, a positive body image is not the state in which an individual is completely satisfied with aspects of body appearance alone [7]. It includes satisfaction with the body's appearance; respect or gratitude for the body; appreciation of the body's function; and protection by judging and processing information associated with ideal attractiveness, which is influenced by society and culture [7,8].

During the perinatal period, body image is influenced by various changes that accompany pregnancy and childbirth. However, the attitude toward changes in the body substantially varies by person, aspect of body image construct, part of the body, and stage of pregnancy and postpartum [9]. For instance, pregnant women experience complex conflicts related to their body image and protect their body image by distinguishing between "fatness" and "pregnancy" [10]. Women may change their focus related to their body image from the aesthetic appearance of the body to the function, while they perceive the pressure of restricting weight gain during pregnancy [11]. Hence, women may adjust their body image by exploring various tactics, which may pose a considerable challenge for some individuals.

Body image is one of the important factors affecting mental health across all ages [12]. It has also been indicated that there are positive associations between body image dissatisfaction and depression during pregnancy [5,13]. A longitudinal study revealed that body dissatisfaction experienced before or during pregnancy is associated with concurrent depressive symptoms or depressive symptoms at the following stage [13], which implies that body image may cause deteriorations in women's mental health throughout the perinatal period. A previous review also concluded that dissatisfaction with body image negatively influences prenatal and postnatal depressive symptoms [5]. Although the adverse influence of body image during pregnancy on antenatal mental health has been investigated, studies focusing on the relationship between the positive side of body image and mental health are scarce among pregnant women. In addition, most studies investigating the association between body image and mental health during the perinatal period have been conducted in Western countries, such as the US, Australia, and Canada [5]. Society and culture play an important role in generating body image and mental health during pregnancy [14]. Therefore, this study aimed to investigate the association between positive body image and mental health during the second trimester of pregnancy among Japanese women.

We hypothesized that higher positive body image would be associated with lower risk of psychological distress and serious mental illness during the second trimester of pregnancy, and explored whether this association would differ based on the accuracy of body size perception.

## Materials and methods

Study design and settings

This was a cross-sectional study using an internet-based questionnaire. Recruitment was conducted in Japan between June and July 2021 through a pregnancy website that required registration. Participants were recruited through a pregnancy information website requiring user registration. This internet-based sampling method may introduce selection bias as participants must have internet access, be registered on the pregnancy website, and be motivated to complete the survey. The participants were asked to answer self-reported online questionnaires in Japanese. All participants read the explanation about the study and provided their consent by submitting their responses. This study was conducted after obtaining approval from the Kyushu University Institutional Review Board for Clinical Research (Approval number: 2021-26, April 21, 2021; Approval number: 22048-00, June 17, 2022).

Participants

Women aged ≥20 years, with a singleton pregnancy, and between 14 and 21 weeks of gestation were recruited. Those who were unable to answer a self-report questionnaire in Japanese were excluded. As a result of recruitment conducted with a target of 500 participants, a total of 520 women answered a pregnancy questionnaire.

Variables and measurements

The Body Appreciation Scale-2 (BAS-2) was used to evaluate body image. It is an instrument to assess body image from a positive aspect, such as approval and acceptance of individuals' bodies [15]. Each of the 10 items is rated from 1 (never) to 5 (always). The mean score was calculated as a continuous variable. A higher average score indicates a better body image. The construct validity and reliability of the Japanese version of the BAS-2 have been confirmed [16]. Cronbach's alpha and test-retest reliability among women were 0.92 and 0.94, respectively [16]. Permission to use the Japanese version of the BAS-2 has been obtained from the researcher [16].

The participants' mental health was evaluated using the Japanese version of the 6-item Kessler Psychological Distress Scale (K6) [17] translated from the original version [18]. It consists of six questions about the situations in the past 30 days, and each of the six items is scored from 0 (none of the time) to 4 (all the time), with total scores ranging from 0 to 24. The Japanese version of the K6 has demonstrated high validity [17]. For analysis purposes, K6 was dichotomized using validated cut-off values. A cut-off value of 4-5 has been proposed to screen for mood/anxiety disorders with a sensitivity of 100% and specificity of 68.7%, indicating its utility in detecting depression or psychological distress. In addition, a value of 12-13 demonstrated a sensitivity of 64.7% and specificity of 97.3%, which suggests that it may be suitable for identifying serious mental illness [19].

Sociodemographic variables included age, pre-pregnancy body mass index (BMI), parity, education level, working status, and cohabitation with a husband. The history of serious illness or operation and sleep duration were collected as health-related variables.

Misperception in body weight was assessed based on participants' self-evaluation of body perception before pregnancy and pre-pregnancy BMI. The participants were categorized into the following groups: "underestimated group" (those who perceived their body size as thin with an actual BMI of >18.5, or those who perceived their body size as average with a BMI of ≥25.0). The "correctly perceived group" included those who perceived their body size as thin with a BMI of <18.5, those who perceived their body size as average with a BMI of 18.5-24.9, or those who perceived their body size as fat with a BMI of ≥25.0. Additionally, the "overestimated group" included those who perceived their body size as average with a BMI of <18.5, or those who perceived their body size as fat with a BMI of <25.0.

Statistical methods

The mean, standard deviation, frequency, and percentage were used to describe the participants' characteristics. Univariable logistic regression was used to explore the bivariate relationship between the variables of participants' characteristics and mental health assessed by the K6. Cut-off scores of 4-5 and 12-13 were used to evaluate factors associated with psychological distress and serious mental illness. Finally, multivariable logistic regression analysis was conducted for K6 cut-off scores of 4-5 and 12-13. Age, pre-pregnancy BMI, parity, history of serious illness or operation, education level, working status, cohabitation with a husband, sleep duration, and gaps in the perception of individuals' shape were adjusted for the models. Misperception between perceived and actual body shape was included in the analysis instead of body perception answered by the participants, since it may be associated with lower psychological well-being, such as lower happiness, higher stress, and even higher suicidal ideation or planning [20]. Additionally, subgroup analysis using univariable logistic regression was performed depending on the misperception of body weight. Multivariable logistic regression was not conducted due to the sample size.

Although a formal a priori sample size calculation was not performed, post hoc assessment of sample adequacy was conducted using the events-per-variable rule for logistic regression. Given an event rate of 59.2% for psychological distress and nine explanatory variables, the available sample size was considered sufficient for the planned analyses. For serious mental illness, the event rate was 14.3%, resulting in a smaller number of outcome events per variable. Accordingly, the analysis should be interpreted with caution. Multivariable logistic regression used the enter method. Model diagnostics were performed, including multicollinearity assessment using variance inflation factors (VIFs) and the Hosmer-Lemeshow goodness-of-fit test. Missing data were minimal in this study. Of the 520 initial responses, 52 (10.0%) were excluded due to incoherent responses (implausible due dates or gestational weeks). Among the remaining 468 participants, complete data were available for BAS-2 and K6. For pre-pregnancy BMI, three participants (0.6%) had missing data. These three participants were also treated as missing for the related variables, body perception, and misperception of body shape. For working status variables, 16 participants (3.4%) had missing data. For cohabitation with a husband, one participant (0.2%) had missing data. After calculating the descriptive statistics, complete-case analysis was applied. For the univariable logistic regression, complete cases for each pair of variables were used, whereas for the multivariable logistic regression, only cases with complete data on all included variables were analyzed.

A p-value of less than 0.05 indicated a statistically significant difference. Statistical analysis was performed using IBM SPSS Statistics for Windows, Version 26 (Released 2018; IBM Corp., Armonk, New York, United States).

## Results

After excluding 52 women with incoherent responses, such as implausible due dates and gestational weeks, 468 pregnant women who answered the online survey were included in the analysis. The internal consistency of the BAS-2 was excellent, with a Cronbach's alpha value of 0.93. Also, Cronbach's alpha of the K6 in our study was 0.89. Table 1 presents the sociodemographic and clinical characteristics of the participants. The mean score of the BAS-2 was 2.8 (standard deviation: 0.8). The number of women who scored above the K6 cut-off values of 5 and 13 were 277 (59.2%) and 67 (14.3%), respectively. In terms of weight perception, all 46 women who underestimated their shape had a BMI of 18.5-24.9. Among 312 women who properly perceived their body size, the number of women with a BMI of <18.5, 18.5-24.9, and >24.9 was 71, 202, and 39, respectively. Finally, 21 women with a BMI of <18.5 and 86 women with a BMI of 18.5-24.9 overestimated their body size.

**Table 1:** Sociodemographic and clinical characteristics of the participants. BAS-2: Body Appreciation Scales-2; BMI: body mass index; K6: 6-item Kessler Psychological Distress Scale; SD: standard deviation

Variable	Mean ± SD or n (%)
Age (n = 468)	31.0 ± 4.2
Pre-pregnancy BMI (n = 465)	20.7 ± 3.0
Sleep duration (n = 468)	7.3 ± 1.0
BAS-2 (n = 468)	2.8 ± 0.8
Pre-pregnancy BMI	
<18.4	92 (19.8)
18.5-24.9	334 (71.8)
24.5>	39 (8.4)
Parity	
Primiparity	279 (59.6)
Multiparity	189 (40.4)
History of serious illness or operation	
Yes	52 (11.1)
No	416 (88.9)
Education level	
High school or less	86 (18.4)
Associate’s degree	149 (31.8)
Bachelor’s or higher degree	233 (49.8)
Working status	
Unemployed	128 (28.3)
Part-time	61 (13.5)
Full-time	263 (58.2)
Cohabitation with a husband	
Yes	455 (97.4)
No	12 (2.6)
Body perception	
Thin	117 (25.2)
Average	222 (47.7)
Fat	126 (27.1)
Misperception in body shape	
Underestimated	46 (9.9)
Properly perceived	312 (67.1)
Overestimated	107 (23.0)
K6 cut-off value of 5	
<5	191 (40.8)
≥5	277 (59.2)
K6 cut-off value of 13	
<13	401 (85.7)
≥13	67 (14.3)

Table 2 lists the results of univariable logistic regression analysis. With a K6 cut-off value of 5, higher scores in BAS-2 were significantly associated with lower risk of psychological distress (odds ratio (OR): 0.68, 95% confidence interval (CI): 0.54-0.86, p = 0.001). When serious mental illness was assessed with a cut-off value of 13, a higher score in BAS-2 predicted a lower risk of serious mental illness (OR: 0.60, 95% CI: 0.43-0.83, p = 0.002).

**Table 2:** Univariable logistic regression for K6 cut-off score of 5 and 13. BAS-2: Body Appreciation Scales-2; BMI: body mass index; CI: confidence interval; K6: 6-item Kessler Psychological Distress Scale; OR: odds ratio; SD: standard deviation

Variable	K6 <5	K6 ≥5	OR (95% CI)	p-value	K6 <13	K6 ≥13	OR (95% CI)	p-value
	Mean ± SD or n (%)	Mean ± SD or n (%)			Mean ± SD or n (%)	Mean ± SD or n (%)		
BAS-2 (n = 468)	2.9 ± 0.9	2.7 ± 0.8	0.68 (0.54-0.86)	0.001	2.8 ± 0.8	2.5 ± 0.8	0.60 (0.43-0.83)	0.002
Misperception in body shape (n = 465)								
Underestimated	19 (9.9)	27 (9.9)	0.96 (0.51-1.81)	0.906	40 (10.0)	6 (9.1)	0.94 (0.38-2.35)	0.892
Properly perceived	126 (66.0)	186 (67.9)	Reference	-	269 (67.4)	43 (65.2)	Reference	-
Overestimated	46 (24.1)	61 (22.3)	0.90 (0.58-1.40)	0.636	90 (22.6)	17 (25.8)	1.18 (0.64-2.18)	0.592

Table 3 summarizes the results of the multivariable logistic regression analysis to evaluate the association between body image and mental illness. Model diagnostics showed no problematic multicollinearity (all VIFs <2.0). The Hosmer-Lemeshow test showed adequate fit for K6 ≥5 (χ^2^ = 5.09, p = 0.75) and K6 ≥ 13 (χ^2^ = 9.33, p = 0.315) models. Nagelkerke R^2^ values were 0.106 for K6 ≥ 5 and 0.102 for K6 ≥ 13 models. Even after adjusting for age, pre-pregnancy BMI, parity, history of serious illness or operation, education level, working status, cohabitation with a husband, sleep duration, and misperception in the individuals' shape, those who had a more positive body image were less likely to have psychological distress as assessed using the K6 cut-off value of 5 (adjusted OR: 0.65, 95% CI: 0.50-0.84, p = 0.001). Positive body image also predicted a low risk of serious mental illness assessed using the K6 cut-off value of 13 (adjusted OR: 0.68, 95% CI: 0.47-0.97, p = 0.034). The adjusted ORs suggest moderate associations. For the serious mental illness outcome, the CI (0.47-0.97) is borderline significant, reflecting limited statistical precision due to the smaller number of events (n = 67) relative to the number of predictors, resulting in approximately seven events per predictor, below the commonly recommended guideline of 10 events per predictor.

**Table 3:** Multivariable logistic regression for K6 cut-off score of 5 and 13. Age, pre-pregnancy BMI, parity, history of serious illness or operation, education level, working status, cohabitation with a husband, sleep duration, and misperception in body shape were adjusted in the model. aOR: adjusted odds ratio; CI: confidence interval; BAS-2: Body Appreciation Scales-2; K6: 6-item Kessler Psychological Distress Scale

Variable	K6 cut-off value of 5 (n = 448)			K6 cut-off value of 13 (n = 448)		
	aOR	95% CI	p-value	aOR	95% CI	p-value
BAS-2	0.65	0.50-0.84	0.001	0.68	0.47-0.97	0.034
Misperception in body shape			0.790			0.968
Underestimated	0.89	0.46-1.74	0.739	1.08	0.41-2.84	0.875
Properly perceived	Reference	-	-	Reference	-	-
Overestimated	0.85	0.52-1.39	0.519	1.08	0.56-2.08	0.822

Subgroup analysis

The association between BAS-2 and K6 was explored among the groups of people with and without a mismatch between perceived and actual body shape by univariable logistic regression analysis (Table 4). The results showed that positive body image did not predict psychological distress assessed using the K6 cut-off value of 5 among women who underestimated their body size (OR: 0.71, 95% CI: 0.34-1.48, p = 0.353). In contrast, it was associated with a reduction in the risk among women who properly assessed their size (OR: 0.75, 95% CI: 0.57-0.99, p = 0.044) and those who overestimated it (OR: 0.43, 95% CI: 0.25-0.77, p = 0.004).

**Table 4:** Univariable logistic regression for K6 cut-off score of 5 and 13 among women underestimated, accurately perceived, and overestimated one’s body weight. OR: odds ratio; CI: confidence interval; BAS-2: Body Appreciation Scales-2; K6: 6-item Kessler Psychological Distress Scale

Variable	K6 cut-off value of 5					K6 cut-off value of 13		
	OR		95% CI	p-value		OR	95% CI	p-value
Underestimated (n = 46)								
BAS-2	0.71		0.34-1.48	0.353		0.28	0.08-1.00	0.049
Accurately perceived (n = 312)								
BAS-2	0.75		0.57-0.99	0.044		0.86	0.58-1.26	0.439
Overestimated (n = 107)								
BAS-2	0.43		0.25-0.77	0.004		0.18	0.07-0.45	<0.001

Regarding the risk of serious mental illness as measured using the K6 cut-off value of 13, BAS-2 did not predict psychological health among women properly perceiving their body size (OR: 0.86, 95% CI: 0.58-1.26, p = 0.439). However, body image was substantially associated with mental health among women, with a gap between perceived and actual body size. The ORs were 0.28 (95% CI: 0.08-1.00, p = 0.049) and 0.18 (95% CI: 0.07-0.45, p < 0.001) for the underestimated and overestimated groups, respectively.

## Discussion

This study assessed the relationship between body image and mental health during pregnancy. Individuals with a more positive body image, compared with those who had a lower score using the BAS-2, showed a significantly lower risk of psychological distress and serious mental illness during the second trimester of pregnancy. The average BAS-2 score of 2.8 in this study was higher than the score of 2.5 in female Japanese university students [16], whereas it was lower than the score of 3.2 from a US community sample [15]. It is possible that the physical and psychological changes associated with pregnancy may have contributed to a more positive emotional feeling towards the body among Japanese women. Having a positive attitude towards the body during pregnancy may reduce the conflict that women experience regarding the physical changes [10], thereby possibly contributing to a reduction in psychological stress. According to previous research, women perceive that their pregnant body is incongruent with pre-existing social roles [10], which may lead to psychological stress. Positive feelings towards the body may reduce the incongruence by accepting pregnancy-related changes, which may contribute to alleviating stress in the complex environment during pregnancy.

The results of the positive association between body image and mental health during pregnancy were mostly consistent with those in previous studies [13,21-24]. However, the body image assessment methods in previous studies were different from the one used in our study. For instance, some studies used a scale focusing on body dissatisfaction, or satisfaction with some specific parts of the body, or with the overall appearance of the body [13,21]. A significant association between body image and depressive symptoms was also demonstrated in the studies using a scale with positive and negative aspects of body image [22-24]. In our study, body image was assessed by a scale that focused on appreciation and respect for the body, which are distinct from body dissatisfaction, attitudes toward particular parts of the body, or feelings toward only the appearance of the body. The results of our study added to the evidence regarding this point.

Our study demonstrated that higher positive body image was associated with lower psychological distress. Considering the several adverse effects of psychological distress during pregnancy on the offspring, including lower Apgar score and admission during the neonatal period [25], addressing both serious levels of psychological disorders and low levels of distress is important for improving maternal and child health. Given the findings of our study, improving positive body image may lead to beneficial outcomes in both women and their offspring, not only by preventing particular psychological disorders but also by reducing a low level of psychological distress.

In contrast to previous studies [20,26], the misperception of body shape itself did not predict psychological distress or serious mental illness. In previous studies [20,26], the relationship between women's perception of body size and their mental health was assessed in middle or high school girls and female college students, whereas the participants in our study were pregnant women. One possible reason for this difference might be that, as mental health was assessed in the second trimester of pregnancy, stress related to weight misperception was alleviated as women experienced pregnancy-related physical and psychological changes. Additionally, only a small number of women with a BMI of <18.5 and overestimated body weight participated in the study, which might have affected the results.

In subgroup analysis, positive body image was associated with a reduced risk of psychological distress among people who properly perceived or overestimated their body weight. Also, the association between positive body image and a risk of serious mental illness was substantial among women who overestimated and underestimated their body size. These associations were considerable among women who overestimated their body size. Overestimation of body size is related to the desire to control weight and pressure to lose weight; as a result, it might negatively affect psychological condition [26,27]. It is also associated with dissatisfaction with body weight [26], implying a low level of positive body image. Given the results of previous studies, overestimation of body size might emphasize the effect of a low positive body image on poor mental health. However, differences in body size perception were not directly related to mental health in our study. To understand the effects of body weight misperception, further studies, particularly among pregnant women who overestimate their weight, are needed.

Nonetheless, the results of this study confirmed the importance of positive body image in pregnant women's psychological health. These findings have several clinical implications. Healthcare providers should assess not only BMI and gestational weight gain but also pregnant women's body image and perception of body weight. Brief screening for body image could help identify those at risk of psychological distress, particularly among women who overestimate their body size.

From a public health perspective, these findings should be considered in maternal and child health programs as well as prenatal education. In educational and counseling settings, emphasizing the acceptance of bodily changes and focusing on body function rather than appearance may be considered important.

The current study was novel in that it focused on positive attitudes toward or respect for the body instead of negative aspects of body image. However, our study has some limitations. First, this was a cross-sectional study among pregnant women of 14-21 weeks of gestational age. Therefore, causality cannot be inferred from our findings. While we observed that higher positive body image was associated with lower risk of psychological distress and serious mental illness, the temporal relationship and direction of this association remain unclear. It is possible that positive body image protects against mental health problems, but it is equally plausible that better mental health promotes more positive body image, or that bidirectional relationships exist. The findings of this study were also limited to women in the second trimester of pregnancy. Longitudinal studies are needed to establish temporal precedence and support causal inference. Second, the study used an internet-based questionnaire, which could lead to selection bias. However, the participation of non-pregnant women was restricted because of the use of pregnancy websites requiring registration. Third, multivariable analysis was not performed in the subgroup analysis due to the small sample size. Additional limitations include: we did not assess potential confounders such as detailed socioeconomic indicators or previous history of mental illness. Body perception and BMI were self-reported, which may introduce measurement error. The relatively small number of events for serious mental illness, with nine predictors, results in approximately seven events per predictor, below the guideline of 10 events per predictor. The borderline CI (95% CI: 0.47-0.97) reflects this limited precision, and these results should be interpreted with appropriate caution. While model diagnostics suggest acceptable model fit, replication in larger samples is needed to confirm the stability of these findings for serious mental illness. These limitations should be considered when interpreting the results of our study.

## Conclusions

There was a statistically significant association between positive body image, such as gratitude and a favorable attitude toward individuals' bodies, and a low risk of psychological distress as well as serious mental illness, in pregnant women of 14-21 weeks of gestational age. The misperception of body shape was not associated with psychological distress or serious mental illness. However, there was a possibility that the association between positive body image and women's mental health might be greater among people who overestimated their body weight. Further longitudinal research with larger sample sizes is needed to confirm this finding.

These findings suggest that positive body image may influence improvements in mental health during pregnancy. Clinical staff should be aware not only of women's BMI and weight gain during pregnancy but also their body image and perception of body weight to improve their well-being.
